# Supplementary material for: Oxidative Stress, Glutathione Metabolism, and Liver Regeneration Pathways Are Activated in Hereditary Tyrosinemia Type 1 Mice upon Short-Term Nitisinone Discontinuation
Source: Genes (Basel). 2020 Dec 22;12(1):3. doi: 10.3390/genes12010003 (PMC7822164; doi:10.3390/genes12010003)
Supplement: Supplementary file 1 [file genes-12-00003-s001.pdf]

Supplementary Information

Oxidative Stress, Glutathione Metabolism and Liver Regeneration Pathways are Activated in Hereditary Tyrosinemia Type 1 Mice upon short-term Nitisinone Discontinuation

Haaike Colemonts-Vroninks, Jessie Neuckermans, Lionel Marcelis, Paul Claes, Steven Branson, Georges Casimir, Philippe Goyens, Geert A. Martens, Tamara Vanhaecke and Joery De Kock

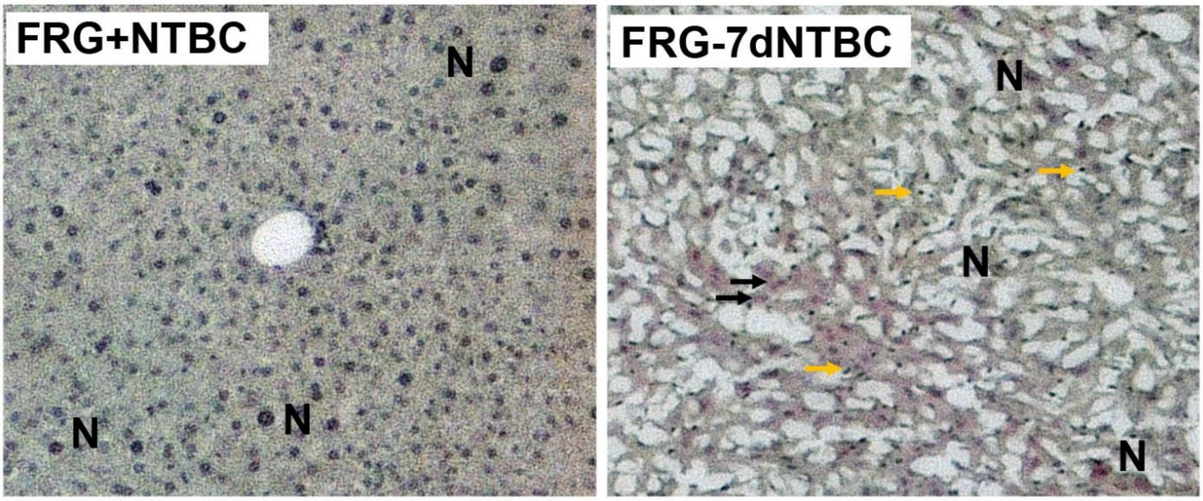

Figure S1: Histopathology confirms presence of severe liver lesions in NTBC-deprived FRG mice. FRG mice continuously treated with NTBC only present mild hepatocellular changes as hepatocytes are enlarged and regularly show large dysmorphic nuclei (N) with several well-formed nucleoli (left panel). In strong contrast, FRG mice at seven days post withdrawal of NTBC present severe hepatocellular changes including necrosis of hepatocytes, enlarged hepatocytes, large dysmorphic nuclei (N), oval-like cell proliferation (black arrow) and infiltration of small lymphoid cells with dark nuclei and scant cytoplasm (yellow arrow) (right panel)

Table S1: Taqman gene expression assays

Gene	Assay-on-Demand ID	Amplicon length (bp)	Source
Afp	Mm00431715_m1	96	AB
Alb	Mm00802090_m1	89	AB
Atf3	Mm00476032_m1	61	AB
Cd24a	Mm00782538_sH	142	AB
Cyr61	Mm00487498_m1	112	AB
Ddr1	Mm01273496_m1	68	AB
Elovl7	Mm00512434_m1	81	AB
Epcam	Mm00493214_m1	95	AB
Gapdh	Mm99999915_g1	109	AB
Ggt1	Mm00492322_m1	98	AB
Glis3	Mm00615386_m1	101	AB
Hmbs	Mm01143545_m1	81	AB
Hspa1a	Mm01159846_s1	121	AB
Krt19	Mm00492980_m1	109	AB
Prom1	Mm01211402_m1	65	AB
Slc7a11	Mm00442530_m1	66	AB
Ubc	Mm02525934_g1	176	AB

* AB: Applied Biosystems

Table S2: Differential gene expression in liver tissue of FRG-7dNTBC *versus* FRG+NTBC mice with 5-fold cut-off and FDR p-value ≤ 0.05. Multiple probe sets were analyzed for the same gene, if present, and listed in the table.

Gene Symbol	Fold change	p-value	FDR p-value	Probe set
Slc7a11	300.44	1.220E-06	6.900E-03	1443536_at
Ggt1	121.39	5.400E-05	1.130E-02	1448485_at
Slc7a11	119.19	3.580E-07	6.400E-03	1420413_at
Hmox1	58.78	1.200E-03	2.320E-02	1448239_at
Slc1a4	47.69	7.870E-05	1.190E-02	1423549_at
Cbr3	46.33	7.630E-05	1.190E-02	1427912_at
Pcdha1; Pcdha10;	44.10	7.930E-06	9.500E-03	1420798_s_at
Afp	42.40	5.000E-04	1.750E-02	1416646_at
Mgst2	39.90	3.000E-04	1.590E-02	1452592_at
Lcn2	39.80	1.800E-03	2.710E-02	1427747_a_at
Elovl7	38.07	3.000E-04	1.540E-02	1441891_x_at
G6pd2; G6pdx	36.79	3.900E-03	3.730E-02	1422327_s_at
Cd9	34.71	4.520E-05	1.130E-02	1416066_at
Cdkn1a	34.15	1.120E-05	9.600E-03	1421679_a_at
Cdkn1a	34.05	3.950E-05	1.100E-02	1424638_at
Elovl7	33.75	2.000E-04	1.480E-02	1424098_at
Zmynd12	31.62	4.610E-06	9.100E-03	1459815_at
Tinag	30.61	1.000E-03	2.180E-02	1419314_at
Cd36	29.95	1.000E-03	2.220E-02	1450883_a_at
Elovl7	27.99	2.000E-04	1.440E-02	1424097_at
Ptgr1	27.09	4.750E-05	1.130E-02	1417777_at
Ptgs2	26.40	5.300E-03	4.290E-02	1417262_at
Elovl7	26.30	8.080E-05	1.200E-02	1440354_at
Ly6d	26.02	6.000E-04	1.910E-02	1416930_at
G6pdx	25.32	1.240E-05	9.600E-03	1448354_at
Afp	24.15	2.000E-03	2.820E-02	1416645_a_at
Myom1	23.82	8.480E-05	1.200E-02	1420693_at
Mgst3	23.34	3.400E-03	3.510E-02	1448300_at
Srxn1	23.27	1.010E-05	9.600E-03	1426875_s_at
Slc1a4	22.52	1.450E-05	9.700E-03	1423550_at
Ddit4l	22.07	1.400E-03	2.460E-02	1451751_at
Tnfrsf12a	21.20	5.890E-05	1.130E-02	1418572_x_at
Nt5e	21.16	1.000E-04	1.360E-02	1428547_at
Slc1a4	20.82	8.000E-04	2.050E-02	1456003_a_at
Cidec	20.29	2.000E-04	1.420E-02	1452260_at
Slc1a1	20.14	6.000E-04	1.910E-02	1425415_a_at
Hist1h4h;	20.05	5.400E-03	4.330E-02	1428014_at
Slc16a5	19.83	3.000E-04	1.600E-02	1434473_at
Tnfrsf12a	19.72	1.570E-05	9.900E-03	1418571_at
Mfsd7b	19.43	2.200E-03	2.920E-02	1434424_at
Atf3	19.05	7.000E-04	1.950E-02	1449363_at

Gene Symbol	Fold change	p-value	FDR p-value	Probe set
Pdk4	18.83	6.000E-04	1.850E-02	1417273_at
Afp	18.83	2.000E-03	2.810E-02	1436879_x_at
Srxn1	18.32	8.470E-05	1.200E-02	1425351_at
Elovl7	18.07	6.340E-05	1.130E-02	1440312_at
Hspb1	16.39	5.510E-05	1.130E-02	1425964_x_at
Clcf1	15.69	1.400E-03	2.420E-02	1437270_a_at
S100a10	15.43	2.190E-05	1.010E-02	1416762_at
Nqo1	15.40	7.000E-04	1.920E-02	1423627_at
S100g	15.39	5.000E-04	1.760E-02	1448964_at
Tubb2a	15.32	3.000E-04	1.500E-02	1427347_s_at
Gstm2	15.06	5.560E-05	1.130E-02	1416411_at
Med13	14.90	3.000E-04	1.510E-02	1438725_at
Abcb1b	14.80	9.000E-04	2.140E-02	1418872_at
Ect2	14.40	1.000E-04	1.350E-02	1419513_a_at
Hspb1	14.01	8.830E-05	1.200E-02	1422943_a_at
Bcl2l11	13.94	2.900E-03	3.270E-02	1435449_at
Cachd1	13.75	2.460E-05	1.010E-02	1436030_at
Srxn1	13.55	1.930E-05	1.010E-02	1451680_at
Gpx2	13.40	4.300E-05	1.130E-02	1449279_at
Spink1	13.39	7.000E-04	2.010E-02	1415938_at
Inhbb	13.30	3.190E-06	9.100E-03	1426858_at
Gla	13.27	4.000E-04	1.640E-02	1418248_at
Cachd1	13.24	6.000E-04	1.840E-02	1436031_at
Mt2	13.12	3.000E-04	1.600E-02	1428942_at
Gsr	13.06	1.500E-03	2.500E-02	1421816_at
Tchh	12.96	6.240E-05	1.130E-02	1434425_at
Morc4	12.81	1.000E-04	1.330E-02	1434436_at
Eomes	12.58	4.000E-03	3.790E-02	1435172_at
D16Ertd472e	12.49	2.000E-04	1.400E-02	1424724_a_at
Pcdha1; Pcdha10;	12.26	6.000E-04	1.880E-02	1424341_s_at
Ndrg1	11.88	2.100E-03	2.850E-02	1423413_at
Mecom	11.67	8.000E-04	2.060E-02	1438325_at
Pcna	11.63	8.000E-04	2.030E-02	1417947_at
Ptpn14	11.59	1.100E-03	2.250E-02	1421499_a_at
Klf4	11.56	1.700E-03	2.600E-02	1417394_at
Maoa	11.44	3.000E-04	1.600E-02	1428667_at
Ndrg1	10.96	9.000E-04	2.090E-02	1420760_s_at
Pcdha1; Pcdha10;	10.94	2.000E-04	1.420E-02	1451769_s_at
Nln	10.93	8.000E-04	2.030E-02	1424981_at
Pttg1	10.81	2.400E-03	3.030E-02	1424105_a_at
Ptbp3	10.74	9.270E-05	1.230E-02	1424083_at
Adh7	10.70	8.360E-05	1.200E-02	1421058_at
C920025E04Rik;	10.70	4.000E-04	1.710E-02	1419658_at
Abcc4	10.65	1.000E-03	2.170E-02	1443870_at
Ddit4l	10.29	1.600E-03	2.580E-02	1444139_at
Bcl2l11	10.13	5.800E-03	4.500E-02	1426334_a_at
Gm8465; Ppcs	10.12	2.500E-03	3.060E-02	1417473_a_at

Gene Symbol	Fold change	p-value	FDR p-value	Probe set
Abcb1a	10.06	1.000E-04	1.280E-02	1419758_at
Lgals3	9.92	4.300E-03	3.880E-02	1426808_at
Sptlc2	9.91	3.730E-06	9.100E-03	1460243_at
Pgd	9.84	7.100E-05	1.170E-02	1423706_a_at
Plpp1	9.82	2.400E-03	3.050E-02	1422620_s_at
Plpp1	9.77	2.400E-03	3.010E-02	1422619_at
B930041F14Rik	9.76	4.000E-04	1.730E-02	1438635_x_at
Larp4	9.75	5.000E-04	1.800E-02	1455102_at
Tubb6	9.74	4.000E-04	1.620E-02	1416431_at
Naip2	9.55	1.800E-03	2.660E-02	1460273_a_at
Pkm	9.51	6.980E-05	1.170E-02	1417308_at
Ero1l	9.49	1.090E-05	9.600E-03	1419029_at
Matn2	9.48	1.000E-03	2.180E-02	1455978_a_at
Rhpn2	9.41	2.000E-04	1.420E-02	1431805_a_at
Matn2	9.23	3.000E-04	1.570E-02	1419442_at
Slc39a6	9.12	3.000E-04	1.600E-02	1424674_at
Pdlim7	9.03	2.000E-04	1.440E-02	1428319_at
Tubb4b	9.01	3.910E-05	1.100E-02	1423642_at
Ndrg1	8.97	1.000E-03	2.210E-02	1450976_at
Mllt11	8.96	1.000E-04	1.360E-02	1416313_at
Nid1	8.95	1.000E-04	1.330E-02	1416808_at
Acot10; Acot9	8.94	6.000E-04	1.880E-02	1449968_s_at
Rtn4	8.93	4.500E-03	3.990E-02	1421116_a_at
Eda2r	8.91	8.780E-05	1.200E-02	1440085_at
Pnp; Pnp2	8.91	9.000E-04	2.160E-02	1453299_a_at
Dnajc12	8.79	3.000E-04	1.580E-02	1417441_at
Acot9	8.79	6.000E-04	1.880E-02	1418073_at
Gmfb	8.77	6.190E-06	9.300E-03	1448571_a_at
Net1	8.67	6.400E-03	4.710E-02	1421321_a_at
Nabp1	8.62	1.500E-03	2.480E-02	1460521_a_at
Ndrg1	8.62	1.800E-03	2.690E-02	1456174_x_at
Amn	8.58	2.100E-03	2.870E-02	1417920_at
Ccdc120	8.55	2.000E-04	1.410E-02	1428066_at
Dynll1	8.54	4.590E-05	1.130E-02	1448682_at
Cldn1	8.47	3.000E-04	1.560E-02	1450014_at
Acsl4	8.44	3.000E-04	1.570E-02	1451828_a_at
Dynlt1-ps1;	8.38	5.000E-04	1.820E-02	1453473_a_at
Glis3	8.37	4.000E-04	1.720E-02	1430353_at
Btg3; Gm7334	8.36	7.170E-05	1.170E-02	1449007_at
Chml	8.31	2.000E-04	1.430E-02	1435926_at
Hacd3	8.31	4.500E-03	3.970E-02	1452427_s_at
Ube2d3	8.27	3.600E-03	3.610E-02	1455480_s_at
Id1	8.21	1.000E-04	1.280E-02	1425895_a_at
Sntb2	8.17	1.000E-04	1.330E-02	1436986_at
Cdkn2b	8.15	8.060E-05	1.200E-02	1449152_at
Slc3a2	8.14	3.000E-04	1.580E-02	1425364_a_at
Ces2c; Ces2d-ps	8.12	8.000E-04	2.020E-02	1424245_at

Gene Symbol	Fold change	p-value	FDR p-value	Probe set
Suco	8.06	9.610E-05	1.230E-02	1460573_at
9430069I07Rik	8.05	2.700E-03	3.190E-02	1431672_at
Mkrn1	8.02	5.000E-04	1.800E-02	1455504_a_at
Tstd1	8.02	4.500E-03	3.990E-02	1456169_at
Hsp90aa1	7.97	2.000E-04	1.450E-02	1437497_a_at
Slc48a1	7.95	2.000E-04	1.440E-02	1450409_a_at
Ucp2	7.91	5.000E-04	1.740E-02	1459740_s_at
Gipc2	7.85	6.000E-04	1.840E-02	1417178_at
Agfg1	7.81	3.000E-03	3.310E-02	1452237_at
Rcan1	7.81	2.000E-04	1.420E-02	1416600_a_at
St3gal6	7.73	1.600E-03	2.560E-02	1449078_at
Nabp1	7.72	7.110E-05	1.170E-02	1455679_at
Fem1b	7.72	3.000E-04	1.600E-02	1418323_at
Tardbp	7.68	6.960E-05	1.170E-02	1434419_s_at
Pgm1	7.63	3.540E-05	1.070E-02	1453283_at
Tpm1	7.59	9.000E-04	2.090E-02	1423049_a_at
Slc48a1	7.57	4.670E-06	9.100E-03	1450410_a_at
Serpinb1b	7.54	6.000E-04	1.910E-02	1426318_at
Nedd9	7.53	5.000E-04	1.790E-02	1422818_at
Agfg1	7.51	2.000E-04	1.480E-02	1426923_at
Slc1a1	7.46	2.000E-04	1.380E-02	1448299_at
Meig1	7.44	2.000E-04	1.490E-02	1423410_at
Psph	7.42	5.000E-04	1.820E-02	1415673_at
Tpr	7.36	4.000E-04	1.710E-02	1456651_a_at
Dppa5a	7.31	2.000E-04	1.430E-02	1416552_at
Psrc1	7.30	2.000E-04	1.410E-02	1417323_at
Mkrn1	7.27	5.000E-04	1.840E-02	1451425_a_at
Tmem62	7.24	2.560E-05	1.010E-02	1455350_at
Lysmd2	7.22	7.570E-05	1.190E-02	1428626_at
Phtf2	7.21	9.000E-04	2.150E-02	1437637_at
Dusp6	7.18	1.450E-05	9.700E-03	1415834_at
Pdss1	7.11	1.400E-03	2.450E-02	1431893_a_at
Zfand5	7.07	1.000E-04	1.320E-02	1416084_at
Megf9	7.06	3.100E-03	3.360E-02	1433968_a_at
H2-Q2	7.03	5.000E-04	1.790E-02	1418734_at
Smoc2	7.01	4.000E-04	1.670E-02	1415935_at
Nrg4	7.01	7.000E-04	1.960E-02	1421681_at
Tmprss2	6.98	7.120E-05	1.170E-02	1458347_s_at
Prune	6.97	2.090E-05	1.010E-02	1434138_at
Eef1e1	6.96	5.550E-05	1.130E-02	1449044_at
Itgav	6.96	3.700E-03	3.650E-02	1421198_at
Prg4	6.94	4.000E-03	3.790E-02	1449824_at
Smoc2	6.93	1.500E-03	2.520E-02	1431362_a_at
Ifrd1	6.85	3.000E-04	1.550E-02	1416067_at
Ap1s3	6.83	8.000E-04	2.010E-02	1455735_at
Gsr	6.82	9.430E-05	1.230E-02	1421817_at
Lrp11	6.81	1.100E-03	2.270E-02	1433536_at

Gene Symbol	Fold change	p-value	FDR p-value	Probe set
Phlda3	6.78	8.000E-04	2.060E-02	1449002_at
Scoc	6.77	3.800E-03	3.710E-02	1430999_a_at
Fgf21;	6.74	2.600E-03	3.150E-02	1422916_at
Klf6	6.74	1.200E-03	2.350E-02	1427742_a_at
Abcb1a	6.74	1.100E-03	2.240E-02	1419759_at
Rtn4rl2	6.72	4.980E-05	1.130E-02	1439573_at
Rictor	6.69	1.200E-03	2.320E-02	1453775_at
Rnd3	6.67	1.000E-03	2.230E-02	1416701_at
Gm3417; Gm3448;	6.64	8.000E-04	2.050E-02	1421682_a_at
Cbr1	6.64	2.800E-03	3.240E-02	1460196_at
Cyr61	6.64	1.500E-03	2.500E-02	1438133_a_at
Id4	6.61	7.000E-04	1.960E-02	1423259_at
Pgd	6.60	2.000E-03	2.790E-02	1437380_x_at
Mpp5	6.60	1.600E-03	2.570E-02	1450113_at
Nup62	6.59	2.600E-03	3.120E-02	1415926_at
Ccdc86	6.58	2.600E-03	3.100E-02	1454197_a_at
Tubb2a	6.56	3.000E-04	1.520E-02	1427838_at
Fosl2	6.54	2.000E-04	1.400E-02	1437247_at
Gstm5	6.54	1.800E-03	2.710E-02	1416842_at
Aasdhppt	6.53	6.200E-03	4.650E-02	1428757_at
Golim4	6.48	1.600E-03	2.570E-02	1428875_at
Scamp1	6.46	1.700E-03	2.650E-02	1453054_at
Samd4	6.45	8.580E-05	1.200E-02	1424594_at
Hfe	6.43	3.000E-04	1.510E-02	1450702_at
Pop1	6.42	5.000E-03	4.160E-02	1428458_at
Cstb	6.36	2.900E-03	3.280E-02	1422507_at
Gm31718	6.36	5.000E-04	1.790E-02	1442988_at
Hsp90aa1	6.35	1.300E-03	2.350E-02	1426645_at
Pdhx	6.35	2.000E-03	2.820E-02	1456090_at
Vopp1	6.34	4.000E-04	1.640E-02	1451127_at
Pgd	6.30	1.700E-03	2.650E-02	1436771_x_at
Pi4k2b	6.28	2.800E-03	3.220E-02	1420411_a_at
Hist2h3c1	6.28	2.000E-04	1.470E-02	1442051_at
Lonrf3	6.24	3.000E-04	1.570E-02	1429863_at
Cd36	6.24	2.700E-05	1.010E-02	1423166_at
Klf6	6.23	1.000E-04	1.240E-02	1433508_at
Fam234b	6.22	3.000E-04	1.510E-02	1449936_at
Atp13a3	6.22	7.300E-06	9.500E-03	1442145_at
Orm2	6.18	3.300E-03	3.470E-02	1420438_at
Nol8	6.13	8.000E-04	2.010E-02	1452974_at
Gja1	6.13	2.000E-03	2.810E-02	1415800_at
Pphln1	6.13	3.000E-04	1.510E-02	1435766_at
Bche	6.12	4.800E-03	4.120E-02	1437863_at
Lamp3; Ppid	6.10	2.000E-04	1.410E-02	1417057_a_at
Ugp2	6.08	2.000E-04	1.430E-02	1451742_a_at
Ier5	6.07	8.000E-04	2.070E-02	1417612_at
Cyp2b10	6.05	1.800E-03	2.710E-02	1425645_s_at

Gene Symbol	Fold change	p-value	FDR p-value	Probe set
Abhd2	6.02	3.000E-04	1.590E-02	1440447_at
Plaur	6.01	6.000E-04	1.850E-02	1452521_a_at
Sept11	5.99	1.400E-03	2.450E-02	1429234_s_at
Dram1	5.95	1.800E-03	2.710E-02	1424524_at
Klf6	5.95	2.000E-04	1.430E-02	1418280_at
Abhd5	5.93	3.000E-04	1.600E-02	1417566_at
Abhd2	5.93	1.300E-03	2.350E-02	1418661_at
Txlng	5.90	3.000E-04	1.600E-02	1439131_at
Ddit4	5.90	3.200E-03	3.390E-02	1428306_at
Hn1	5.89	1.700E-03	2.620E-02	1416028_a_at
Ear-ps2; Ear1;	5.89	7.670E-05	1.190E-02	1422411_s_at
S100a11	5.89	1.200E-03	2.350E-02	1460351_at
Cyp2b10	5.87	3.900E-03	3.730E-02	1422257_s_at
Zwint	5.84	6.000E-03	4.570E-02	1427540_at
Rhpn2	5.83	5.000E-04	1.770E-02	1434628_a_at
Zwint	5.83	7.200E-03	5.000E-02	1423724_at
Ndfip2	5.82	1.800E-03	2.710E-02	1452066_a_at
Cyp4a31	5.81	6.200E-03	4.640E-02	1424943_at
Clcf1	5.81	1.300E-03	2.360E-02	1437271_at
Nadk2	5.80	3.400E-03	3.490E-02	1453422_a_at
Cd14	5.79	1.800E-03	2.710E-02	1417268_at
Hsdl2	5.78	3.800E-03	3.710E-02	1426857_a_at
Tfrc	5.78	2.000E-03	2.820E-02	1422966_a_at
Smu1	5.76	2.600E-03	3.140E-02	1432042_a_at
Atg12	5.76	2.300E-03	2.970E-02	1451747_a_at
Wasf1	5.71	1.200E-03	2.330E-02	1418545_at
Eaf1	5.71	6.780E-05	1.170E-02	1433555_at
Fbxo30	5.70	4.900E-03	4.140E-02	1453137_at
Tmem43	5.69	1.300E-03	2.420E-02	1426434_at
Tuft1	5.69	7.170E-06	9.500E-03	1416689_at
Gm36839	5.69	1.500E-03	2.510E-02	1440485_at
Utp6	5.65	1.700E-03	2.630E-02	1424500_at
Ypel4	5.65	3.300E-03	3.460E-02	1434501_at
Bag3	5.65	6.000E-04	1.880E-02	1422452_at
Nabp1	5.64	6.800E-05	1.170E-02	1426806_at
Hspa1a	5.62	1.500E-03	2.500E-02	1452388_at
Cdc42se1	5.62	2.500E-03	3.050E-02	1428132_at
Adamts1	5.62	1.700E-03	2.660E-02	1450716_at
Uba3	5.58	6.000E-04	1.880E-02	1431746_a_at
Ctps	5.56	2.000E-04	1.450E-02	1416563_at
Clic4	5.52	7.000E-04	2.010E-02	1423392_at
Nme1	5.52	4.000E-04	1.670E-02	1424110_a_at
Pir	5.51	6.000E-04	1.910E-02	1429001_at
Psmb3	5.50	6.500E-03	4.720E-02	1417052_at
Tgfbr2	5.48	1.000E-04	1.350E-02	1426397_at
4930579G24Rik	5.47	1.500E-03	2.510E-02	1429364_at
BC049762	5.46	2.000E-04	1.450E-02	1436433_at

Gene Symbol	Fold change	p-value	FDR p-value	Probe set
Ppm1h	5.44	8.000E-04	2.060E-02	1455737_at
Ly96	5.41	1.100E-03	2.240E-02	1449874_at
Pramef8	5.40	2.100E-03	2.900E-02	1434566_a_at
Kitl	5.40	4.200E-03	3.880E-02	1415855_at
Greb1l	5.39	4.600E-03	4.020E-02	1439341_at
Adm	5.39	2.000E-04	1.440E-02	1416077_at
Ier3	5.38	1.800E-03	2.680E-02	1419647_a_at
Gm39079	5.36	1.300E-03	2.390E-02	1460052_at
Klf6	5.35	5.000E-04	1.760E-02	1447448_s_at
Zfp54	5.33	1.300E-03	2.350E-02	1419239_at
Agpat5	5.32	1.000E-04	1.330E-02	1453257_at
Scoc	5.31	2.000E-04	1.450E-02	1416267_at
Akr1b7	5.31	6.300E-03	4.650E-02	1423556_at
Ajuba	5.28	2.140E-05	1.010E-02	1421344_a_at
Tmem2	5.28	2.500E-03	3.060E-02	1424711_at
Adk	5.27	9.000E-04	2.090E-02	1449641_at
Pdp1	5.26	1.000E-04	1.290E-02	1434228_at
Ddit4l	5.25	2.900E-03	3.260E-02	1439332_at
Dynll1	5.25	1.300E-03	2.400E-02	1417339_a_at
Dusp8	5.25	1.000E-03	2.230E-02	1418714_at
Acyp2	5.24	9.000E-04	2.090E-02	1427943_at
Lyve1	5.23	9.000E-04	2.090E-02	1429379_at
Slc9a6	5.23	2.600E-03	3.110E-02	1435009_at
Plekha8	5.21	2.000E-04	1.420E-02	1436128_at
Nyx	5.19	3.000E-04	1.540E-02	1446344_at
Nabp1	5.18	7.000E-04	1.940E-02	1430623_s_at
Ccng1	5.16	6.000E-04	1.890E-02	1450016_at
Desi2	5.14	5.790E-06	9.300E-03	1425185_at
Lrrc8b	5.14	6.000E-03	4.560E-02	1437087_at
Txnrd1	5.13	2.300E-03	2.970E-02	1424486_a_at
Nmd3	5.12	1.200E-03	2.310E-02	1448133_at
Cklf	5.12	3.880E-05	1.100E-02	1424495_a_at
Ucp2	5.09	3.000E-04	1.510E-02	1459741_x_at
Cycs	5.08	1.300E-03	2.410E-02	1422483_a_at
Gnai1	5.08	4.300E-03	3.900E-02	1434440_at
Kbtbd8	5.07	4.220E-05	1.130E-02	1433902_at
Nop58	5.06	5.960E-05	1.130E-02	1450986_at
Pon3	5.06	3.600E-03	3.620E-02	1419298_at
Arl14ep	5.05	4.000E-04	1.670E-02	1459902_at
Riok2	5.04	2.300E-03	2.970E-02	1423481_at
Uchl3; Uchl4	5.02	1.900E-03	2.740E-02	1449855_s_at
Tuba3a; Tuba3b	5.02	2.900E-03	3.270E-02	1416311_s_at
Mpp1	5.01	2.000E-04	1.430E-02	1450919_at
Entpd8	-5.02	3.000E-04	1.530E-02	1429550_at
Pisd-ps3	-5.02	9.640E-05	1.230E-02	1453145_at
Slco2b1	-5.04	7.630E-05	1.190E-02	1433933_s_at
Srd5a1	-5.05	6.900E-03	4.860E-02	1454649_at

Gene Symbol	Fold change	p-value	FDR p-value	Probe set
Foxp2	-5.07	4.000E-04	1.710E-02	1438231_at
Slc16a2	-5.08	3.000E-04	1.610E-02	1418446_at
Pltp	-5.11	2.500E-03	3.090E-02	1456424_s_at
4930523C07Rik	-5.12	3.040E-05	1.020E-02	1459962_at
Nrbp2	-5.12	7.720E-05	1.190E-02	1424544_at
Zfp148	-5.12	2.400E-03	3.030E-02	1436217_at
Ppp6r3	-5.13	5.900E-03	4.540E-02	1439161_at
Klf12	-5.14	1.000E-04	1.360E-02	1439847_s_at
Smim22	-5.16	3.700E-03	3.650E-02	1439560_x_at
LOC102635783	-5.16	6.800E-03	4.840E-02	1457227_at
Faah	-5.16	4.000E-04	1.690E-02	1434091_at
Il6ra	-5.19	3.300E-03	3.440E-02	1452416_at
Enpp3	-5.21	2.900E-03	3.260E-02	1439260_a_at
Rab17	-5.21	3.260E-05	1.040E-02	1422178_a_at
Ubr2	-5.21	2.000E-04	1.450E-02	1429515_at
Acacb	-5.25	1.000E-04	1.350E-02	1427052_at
Psmd9	-5.25	1.800E-03	2.700E-02	1447670_at
Gm39475	-5.27	1.100E-03	2.290E-02	1444518_at
Epb41l4b	-5.30	4.000E-04	1.640E-02	1418294_at
Fasn	-5.33	7.930E-05	1.190E-02	1423828_at
A530079E22Rik	-5.34	1.000E-04	1.330E-02	1438863_at
Marf1	-5.36	7.000E-04	1.960E-02	1459879_at
Camkk2	-5.36	1.000E-04	1.320E-02	1424474_a_at
Ttc39c	-5.40	9.000E-04	2.090E-02	1426223_at
Sptbn2	-5.40	3.000E-04	1.510E-02	1452269_at
Klf13	-5.41	3.370E-05	1.040E-02	1432543_a_at
Mmp15	-5.43	2.000E-04	1.430E-02	1422597_at
Pisd-ps3	-5.45	5.000E-04	1.740E-02	1453144_at
1500017E21Rik	-5.47	9.000E-04	2.090E-02	1438596_at
Spata2l	-5.49	2.600E-03	3.120E-02	1428338_at
Nmrk1	-5.51	2.300E-03	2.970E-02	1448048_at
Nfia	-5.59	1.600E-03	2.570E-02	1456087_at
Pdk1	-5.60	1.900E-03	2.740E-02	1423747_a_at
Rbmxl2	-5.69	1.300E-03	2.350E-02	1429343_at
Tert	-5.71	9.000E-04	2.100E-02	1456941_at
C2	-5.83	4.000E-04	1.670E-02	1457664_x_at
Col27a1	-5.85	3.500E-03	3.550E-02	1453191_at
Ppm1k	-5.87	2.000E-04	1.450E-02	1452973_at
Uroc1	-5.89	6.300E-03	4.660E-02	1425003_at
Afmid	-5.91	3.000E-04	1.570E-02	1428885_at
Afmid	-5.91	4.000E-04	1.640E-02	1452944_at
Usp2	-5.93	2.600E-03	3.130E-02	1417168_a_at
Agfg2	-5.94	2.300E-03	2.990E-02	1425362_at
Ppm1a	-5.96	1.200E-03	2.290E-02	1452989_at
Slc17a2	-6.03	9.000E-04	2.090E-02	1425034_at
Mug-ps1; Mug1;	-6.05	2.000E-04	1.400E-02	1448854_s_at
Hal	-6.07	9.000E-04	2.140E-02	1418645_at

Gene Symbol	Fold change	p-value	FDR p-value	Probe set
Itih1	-6.08	9.210E-05	1.220E-02	1417973_at
Ccl27a; Ccl27b;	-6.11	3.000E-04	1.540E-02	1434962_x_at
Tenm3	-6.12	2.500E-03	3.060E-02	1429178_at
Map2k6	-6.17	7.800E-05	1.190E-02	1441482_at
Tmem30a	-6.18	2.000E-03	2.800E-02	1448340_at
Nsmf	-6.21	6.000E-04	1.910E-02	1436959_x_at
Cyp2c44	-6.21	2.800E-03	3.230E-02	1424576_s_at
Neb	-6.24	4.000E-04	1.730E-02	1435355_at
Map2k6	-6.26	3.000E-04	1.530E-02	1442725_at
Zfp467	-6.31	9.000E-04	2.090E-02	1441727_s_at
Zfand4	-6.31	1.000E-04	1.370E-02	1429642_at
Col27a1	-6.33	2.000E-03	2.810E-02	1429549_at
Aifm3	-6.33	6.000E-04	1.870E-02	1434742_s_at
C1s1	-6.37	1.000E-04	1.320E-02	1424041_s_at
Cpn2	-6.38	1.400E-03	2.450E-02	1427459_at
Per3	-6.45	3.000E-04	1.600E-02	1421087_at
Hmgcs2	-6.51	6.000E-04	1.880E-02	1431833_a_at
Slc26a1	-6.53	4.300E-03	3.880E-02	1451239_a_at
Pcyt2	-6.56	2.630E-05	1.010E-02	1420493_a_at
Fads1	-6.59	5.100E-03	4.220E-02	1423680_at
Fads2	-6.64	2.600E-03	3.110E-02	1443838_x_at
Dpys	-6.69	2.600E-03	3.100E-02	1425689_at
Lrrc3	-6.74	3.520E-05	1.070E-02	1431251_at
Cadps2	-6.75	8.730E-05	1.200E-02	1451499_at
Msmo1	-6.77	4.000E-04	1.680E-02	1459627_at
B3galt1	-6.77	5.400E-03	4.330E-02	1441396_at
Igf1	-6.78	1.930E-05	1.010E-02	1419519_at
Srd5a1	-6.80	3.700E-03	3.640E-02	1438699_at
Fn3k	-6.81	1.600E-03	2.530E-02	1418311_at
Hykk	-6.83	2.700E-03	3.170E-02	1435691_at
Car14	-6.88	9.410E-05	1.230E-02	1450725_s_at
Ranbp3l	-6.89	1.100E-03	2.290E-02	1443921_at
Hgfac	-6.92	2.000E-04	1.440E-02	1418405_at
Mmp15	-6.92	3.690E-05	1.080E-02	1437462_x_at
Rtp3	-6.94	4.000E-04	1.680E-02	1452472_at
Mbl1	-6.99	5.280E-05	1.130E-02	1419578_at
Cnot6l	-7.07	1.000E-03	2.180E-02	1434311_at
Csad	-7.10	2.600E-03	3.120E-02	1427981_a_at
Apol7a	-7.11	2.690E-05	1.010E-02	1453080_at
Clec2d	-7.17	4.000E-04	1.670E-02	1419477_at
Tk1	-7.17	3.000E-04	1.570E-02	1416258_at
Luc7l2	-7.18	3.600E-03	3.580E-02	1436766_at
Nfia	-7.21	4.000E-04	1.710E-02	1438236_at
Cyp2j9	-7.24	1.900E-03	2.740E-02	1424677_at
Mug1; Mug2	-7.25	2.500E-03	3.070E-02	1417835_at
Ptprd	-7.33	1.250E-05	9.600E-03	1429052_at
Rsrp1	-7.39	4.100E-03	3.830E-02	1435357_at

Gene Symbol	Fold change	p-value	FDR p-value	Probe set
Sema4g	-7.44	1.200E-03	2.340E-02	1449202_at
Kcnt2	-7.53	7.000E-04	1.920E-02	1459971_at
Prodh	-7.54	1.000E-04	1.360E-02	1417629_at
Adamts7	-7.58	4.000E-04	1.690E-02	1452339_at
Per3	-7.59	2.000E-04	1.480E-02	1442243_at
Eef2k	-7.62	2.000E-04	1.400E-02	1437829_s_at
Proz	-7.63	3.000E-04	1.570E-02	1450201_at
Slco1b2	-7.63	5.540E-05	1.130E-02	1449394_at
Aspdh	-7.64	2.000E-04	1.410E-02	1425117_at
Clmn	-7.70	8.820E-05	1.200E-02	1439117_at
Chn2	-7.71	1.300E-03	2.400E-02	1428573_at
Slc1a2	-7.87	4.000E-04	1.730E-02	1438194_at
Nfix	-7.87	2.500E-05	1.010E-02	1436364_x_at
Klf12	-7.88	4.000E-04	1.650E-02	1455521_at
Hsd3b2; Hsd3b3;	-7.95	6.200E-03	4.630E-02	1460232_s_at
Ttc39c	-7.97	5.790E-05	1.130E-02	1441380_at
Slco1b2	-7.97	3.000E-04	1.600E-02	1452494_s_at
Grem2	-8.10	9.000E-04	2.090E-02	1418492_at
Mup1; Mup10;	-8.10	1.400E-03	2.480E-02	1434110_x_at
Lrg1	-8.15	5.300E-03	4.300E-02	1417290_at
4930523C07Rik	-8.21	6.150E-05	1.130E-02	1456446_at
Prok1	-8.29	3.600E-03	3.590E-02	1443505_at
F7	-8.44	2.000E-04	1.490E-02	1419321_at
Fndc4	-8.45	2.000E-04	1.400E-02	1431226_a_at
Pklr	-8.60	5.000E-04	1.740E-02	1438711_at
Cyp4f14	-8.70	9.000E-04	2.100E-02	1419559_at
Slc22a30	-8.72	2.000E-04	1.480E-02	1436162_at
Amdhd1	-8.74	2.000E-04	1.440E-02	1447380_at
Aox3	-8.76	6.200E-03	4.620E-02	1418858_at
Dclk3	-8.77	6.000E-04	1.910E-02	1436532_at
Nfix	-8.79	7.170E-05	1.170E-02	1436363_a_at
Hdac11	-8.83	8.150E-05	1.200E-02	1454803_a_at
2310067E19Rik	-8.83	4.300E-03	3.890E-02	1438072_at
Rnf152	-8.90	2.000E-04	1.400E-02	1439887_at
Enho	-8.91	2.800E-03	3.220E-02	1452893_s_at
Etnppl	-8.96	3.100E-03	3.360E-02	1431406_at
Masp2	-9.01	1.000E-04	1.360E-02	1451759_at
1700023H06Rik	-9.23	6.000E-04	1.840E-02	1459253_at
Mcm10	-9.29	4.000E-04	1.630E-02	1433408_a_at
Lrp1	-9.33	4.400E-03	3.940E-02	1442849_at
Abcc6	-9.35	4.870E-05	1.130E-02	1421212_at
Cyp2d13	-9.49	4.000E-04	1.660E-02	1431803_at
Scd1	-9.50	2.200E-03	2.920E-02	1415964_at
Mup1; Mup10;	-9.50	5.100E-03	4.230E-02	1426154_s_at
Slc26a1	-9.54	2.000E-04	1.410E-02	1458327_x_at
Per3	-9.68	5.000E-04	1.800E-02	1441445_at
Cml2	-9.69	4.300E-03	3.910E-02	1455232_at

Gene Symbol	Fold change	p-value	FDR p-value	Probe set
Leap2	-9.77	1.000E-03	2.220E-02	1427480_at
Nrep	-9.90	9.000E-04	2.090E-02	1450839_at
Nfia	-10.09	2.800E-03	3.220E-02	1446990_at
Hdac11	-10.18	3.000E-04	1.560E-02	1451229_at
Gm38679	-10.19	2.000E-03	2.840E-02	1458829_at
Ttc39c	-10.30	1.620E-05	9.900E-03	1446769_at
Ces3a; Ces3b	-10.58	3.000E-04	1.600E-02	1451600_s_at
Bace1	-10.62	1.000E-04	1.350E-02	1455826_a_at
Paqr9	-10.74	2.800E-03	3.240E-02	1455025_at
Usp2	-10.85	2.000E-04	1.450E-02	1417169_at
Dixdc1	-10.94	7.060E-05	1.170E-02	1435207_at
Upp2	-11.01	5.000E-04	1.760E-02	1460059_at
Lect1	-11.04	3.900E-03	3.730E-02	1460258_at
Car5a	-11.27	4.400E-03	3.930E-02	1419525_at
Enho	-11.27	4.630E-05	1.130E-02	1428739_at
Hes6	-11.31	8.370E-05	1.200E-02	1436050_x_at
Fam47e	-11.41	1.000E-04	1.250E-02	1455383_at
Ccl9	-11.52	8.000E-04	2.050E-02	1417936_at
Rgs16	-11.95	1.600E-03	2.550E-02	1455265_a_at
Masp1	-11.98	1.170E-06	6.900E-03	1419677_at
Cyp2d13	-12.12	1.000E-04	1.240E-02	1425365_a_at
Crp	-12.13	2.000E-04	1.410E-02	1421946_at
Sc5d	-12.16	8.000E-04	2.050E-02	1434520_at
Amdhd1	-12.30	4.000E-04	1.620E-02	1427370_at
Igf1	-12.39	3.000E-04	1.530E-02	1434413_at
Nox4	-12.67	6.290E-05	1.130E-02	1419161_a_at
Ces1e	-12.77	5.330E-06	9.300E-03	1419510_at
C8a	-12.86	3.000E-04	1.600E-02	1428012_at
Tcf24	-12.9	4.000E-04	1.670E-02	1439816_at
AW111846	-13.86	5.700E-05	1.130E-02	1445657_at
Bcl6	-14.48	9.000E-04	2.090E-02	1421818_at
Cyp2c37	-14.58	2.000E-04	1.430E-02	1419094_at
Dbp	-15.05	7.450E-05	1.190E-02	1418174_at
C8b	-15.36	6.000E-04	1.870E-02	1427472_a_at
Lin7a	-15.37	5.600E-05	1.130E-02	1456656_at
Egfr	-15.41	8.000E-04	2.060E-02	1435888_at
Slc2a2	-16.13	8.000E-04	2.050E-02	1449067_at
Nlrp6	-16.26	8.000E-04	2.030E-02	1427369_at
Gck	-16.68	1.700E-03	2.610E-02	1425303_at
Lin7a	-16.98	1.360E-05	9.600E-03	1435805_at
Dio1	-17.24	5.900E-03	4.530E-02	1417991_at
Cyp7a1	-18.21	1.000E-04	1.290E-02	1438743_at
Gckr	-19.02	5.570E-05	1.130E-02	1426059_at
Cmah	-19.56	4.000E-04	1.730E-02	1421214_at
Lin7a	-19.66	1.140E-06	6.900E-03	1438450_at
Kcnn2	-19.94	6.580E-05	1.160E-02	1445676_at
Nrep	-20.05	4.000E-04	1.720E-02	1436736_x_at

Gene Symbol	Fold change	p-value	FDR p-value	Probe set
B3galt1	-20.43	8.460E-06	9.500E-03	1455234_at
Paqr9	-20.44	2.620E-05	1.010E-02	1436168_at
Igf1	-20.51	4.590E-05	1.130E-02	1437401_at
Hsd3b2	-21.24	5.500E-03	4.350E-02	1425127_at
Cyp2c50; Cyp2c54	-22.60	8.440E-06	9.500E-03	1418653_at
Car3	-23.09	5.000E-04	1.750E-02	1460256_at
Slco1a1	-23.40	2.600E-03	3.120E-02	1449844_at
Serpina4-ps1	-23.65	3.310E-05	1.040E-02	1444297_at
Cmah	-23.77	2.650E-05	1.010E-02	1436039_at
Dbp	-24.13	1.930E-05	1.010E-02	1438211_s_at
LOC545966	-25.49	3.900E-03	3.720E-02	1448080_at
Paqr9	-25.94	4.000E-04	1.630E-02	1436169_at
C730036E19Rik	-27.29	2.250E-05	1.010E-02	1442612_at
Serpina4-ps1	-28.11	2.710E-05	1.010E-02	1448092_x_at
Cyp2j5	-30.38	7.900E-05	1.190E-02	1417532_at
Pigr	-31.23	1.200E-03	2.350E-02	1450060_at
Mup10; Mup18;	-31.75	2.400E-03	3.030E-02	1430893_at
Klkb1	-32.78	2.070E-05	1.010E-02	1449034_at
Serpina4-ps1	-33.41	2.020E-05	1.010E-02	1444296_a_at
Elovl3	-33.42	8.000E-04	2.020E-02	1420722_at
Sult2a8	-40.92	1.200E-03	2.340E-02	1428981_at
Hsd3b3	-43.67	5.680E-05	1.130E-02	1431916_at
Pigr	-50.16	3.000E-04	1.570E-02	1455490_at
Inmt	-51.09	2.160E-05	1.010E-02	1418697_at
Thrsp	-53.31	2.720E-05	1.010E-02	1422973_a_at
Nox4	-59.66	4.930E-06	9.300E-03	1451827_a_at
Cyp2c54	-73.41	4.000E-04	1.620E-02	1455457_at
Slco1a1	-76.01	3.000E-04	1.510E-02	1420379_at
Thrsp	-100.86	8.430E-05	1.200E-02	1424737_at
Hsd3b5	-171.27	1.200E-03	2.310E-02	1420531_at
Upp2	-193.30	1.000E-04	1.290E-02	1424969_s_at
Upp2	-220.46	1.000E-04	1.240E-02	1451548_at
Car3	-320.73	6.190E-06	9.300E-03	1430584_s_at
Car3	-368.42	3.820E-06	9.100E-03	1453588_at

Table S3: Upstream regulator prediction in FRG-7dNTBC *versus* FRG+NTBC liver tissue

Upstream Regulator	Expr Fold Change	Molecule Type	Predicted Activation State	Activation z-score	p-value of overlap
Tnf	1.27	cytokine	Activated	2.521	2.09E-17
Il1b	-1.35	cytokine	Activated	2.429	7.21E-11
Ifng	-1.10	cytokine	Activated	2.329	6.60E-06
Ccl5	-1.06	cytokine	Activated	2.000	1.56E-01
Il1rn	1.52	cytokine	Inhibited	-2.646	2.21E-02
Ptgs2	26.40	enzyme	Activated	2.886	2.09E-02
Elovl5	-3.23	enzyme	Activated	2.643	1.99E-10
Gna12	-1.49	enzyme	Inhibited	-2.219	1.10E-02
Fbxl5	-1.04	enzyme	Inhibited	-2.224	4.60E-07
Cat	-1.37	enzyme	Inhibited	-2.401	2.06E-03
Dicer1	-2.75	enzyme	Inhibited	-3.274	1.54E-02
Gsr	13.06	enzyme	Inhibited	-3.359	5.36E-14
Txnrd1	5.13	enzyme	Inhibited	-3.364	1.59E-12
Acox1	-1.28	enzyme	Inhibited	-4.747	2.01E-17
Tgfb1	-1.21	growth factor	Activated	3.291	1.82E-13
Angpt2	1.66	growth factor	Activated	3.256	1.97E-03
Agt	-2.39	growth factor	Activated	2.772	2.26E-07
Fgf19	-1.12	growth factor	Activated	2.731	1.56E-12
Bmp4	-1.82	growth factor	Activated	2.728	9.24E-02
Bmp6	2.07	growth factor	Activated	2.551	1.55E-03
Jag1	2.61	growth factor	Activated	2.414	3.92E-03
Egf	-1.18	growth factor	Activated	2.357	6.93E-08
Hgf	2.98	growth factor	Activated	2.350	4.40E-04
Vegfa	-1.35	growth factor	Activated	2.219	8.24E-06
Prkcd	1.19	kinase	Activated	3.098	8.14E-03
Raf1	2.65	kinase	Activated	2.970	2.39E-05
Map2k1	2.73	kinase	Activated	2.756	2.25E-05
Map3k1	-1.11	kinase	Activated	2.410	3.92E-03
Mapk7	1.17	kinase	Activated	2.390	2.31E-03
Fgfr2	-1.56	kinase	Activated	2.226	2.85E-04
Tgfbr2	5.48	kinase	Activated	2.154	9.98E-03
Flt1	1.40	kinase	Activated	2.000	1.67E-01
Pak2	1.84	kinase	Inhibited	-2.236	4.04E-03
Cdk19	-1.34	kinase	Inhibited	-2.714	3.67E-06
Nr1i2	-1.58	ligand-dependent nuclear receptor	Activated	3.131	4.35E-14
Esrrg	-1.29	ligand-dependent nuclear receptor	Activated	2.197	4.17E-02
Ppara	-4.93	ligand-dependent nuclear receptor	Activated	2.173	6.09E-22
Ppard	2.38	ligand-dependent nuclear receptor	Inhibited	-2.019	6.24E-08
Safb	-1.31	other	Inhibited	-2.000	5.41E-02
Tsc2	-1.13	other	Inhibited	-2.214	7.71E-05

1810019D21Rik	-1.35	other	**Inhibited**	-2.449	8.04E-03
Cish	-1.21	other	**Inhibited**	-2.646	4.74E-03
F2	-1.46	peptidase	**Activated**	3.300	6.01E-07
F7	-8.44	peptidase	**Activated**	2.213	5.74E-03
Psmb11	-1.15	peptidase	**Inhibited**	-2.157	1.70E-02
Nfe2l2	3.14	transcription regulator	**Activated**	4.563	1.03E-12
Creb1	3.99	transcription regulator	**Activated**	3.557	3.69E-04
Klf4	11.56	transcription regulator	**Activated**	2.952	7.08E-03
Myc	3.69	transcription regulator	**Activated**	2.931	6.46E-11
Kdm3a	1.52	transcription regulator	**Activated**	2.804	6.79E-06
Pou5f1	-1.13	transcription regulator	**Activated**	2.745	7.64E-02
Arnt	1.66	transcription regulator	**Activated**	2.743	9.75E-05
Tp53	3.03	transcription regulator	**Activated**	2.738	2.15E-07
Foxo3	-1.40	transcription regulator	**Activated**	2.569	3.29E-07
Rela	-1.17	transcription regulator	**Activated**	2.568	5.61E-04
Ets1	2.64	transcription regulator	**Activated**	2.423	1.52E-02
Junb	-1.21	transcription regulator	**Activated**	2.412	1.12E-01
Smad4	-2.99	transcription regulator	**Activated**	2.305	1.39E-02
Twist2	-1.21	transcription regulator	**Activated**	2.219	4.04E-03
Ybx1	1.21	transcription regulator	**Activated**	2.219	4.01E-05
Smad2	1.33	transcription regulator	**Activated**	2.207	1.51E-02
Sp1	-1.46	transcription regulator	**Activated**	2.151	3.80E-07
Foxa2	-1.10	transcription regulator	**Activated**	2.146	7.32E-07
Atf4	1.36	transcription regulator	**Activated**	2.034	6.89E-09
Xbp1	-1.65	transcription regulator	**Activated**	2.034	1.00E+00
Smad1	1.70	transcription regulator	**Activated**	2.000	3.85E-02
Ppp1r13l	1.35	transcription regulator	**Activated**	2.000	2.11E-03
Irf3	-1.54	transcription regulator	**Activated**	2.000	1.00E+00
Elf4	-1.28	transcription regulator	**Activated**	2.000	1.02E-02
Spib	-1.05	transcription regulator	**Activated**	2.000	1.85E-01
Ep300	-1.11	transcription regulator	**Activated**	2.000	3.25E-05
Gmnn	-1.05	transcription regulator	**Inhibited**	-2.000	9.78E-02
Tfap4	-1.73	transcription regulator	**Inhibited**	-2.000	2.58E-02
Sox3	-1.26	transcription regulator	**Inhibited**	-2.000	2.09E-01
Tcf7l2	-1.28	transcription regulator	**Inhibited**	-2.111	1.59E-01
Gata3	1.33	transcription regulator	**Inhibited**	-2.122	1.39E-01
Ncor1	1.68	transcription regulator	**Inhibited**	-2.138	1.26E-05
Bcl6	-14.48	transcription regulator	**Inhibited**	-2.144	3.16E-01
Hnf1a	1.03	transcription regulator	**Inhibited**	-2.744	5.06E-08
Stat5b	-1.80	transcription regulator	**Inhibited**	-3.698	1.42E-11
Tlr9	-1.11	transmembrane receptor	**Activated**	2.750	1.96E-01
Tlr4	1.80	transmembrane receptor	**Activated**	2.442	1.49E-01
Ucp1	-1.11	transporter	**Activated**	2.541	3.55E-04
Syvn1	1.79	transporter	**Activated**	2.530	5.84E-03
Slc16a2	-5.08	transporter	**Activated**	2.236	2.38E-03
Bcap31	2.30	transporter	**Activated**	2.000	5.99E-03
Atp7b	-1.12	transporter	**Inhibited**	-2.646	1.59E-04
Tfrc	5.78	transporter	**Inhibited**	-2.714	6.89E-06
